# Coupling Euler–Euler and Microkinetic Modeling for the Simulation of Fluidized Bed Reactors: an Application to the Oxidative Coupling of Methane

**DOI:** 10.1021/acs.iecr.0c05845

**Published:** 2021-02-12

**Authors:** Daniele Micale, Riccardo Uglietti, Mauro Bracconi, Matteo Maestri

**Affiliations:** † Laboratory of Catalysis and Catalytic Processes, Dipartimento di Energia, Politecnico di Milano, via La Masa 34, 20156 Milano, Italy

## Abstract

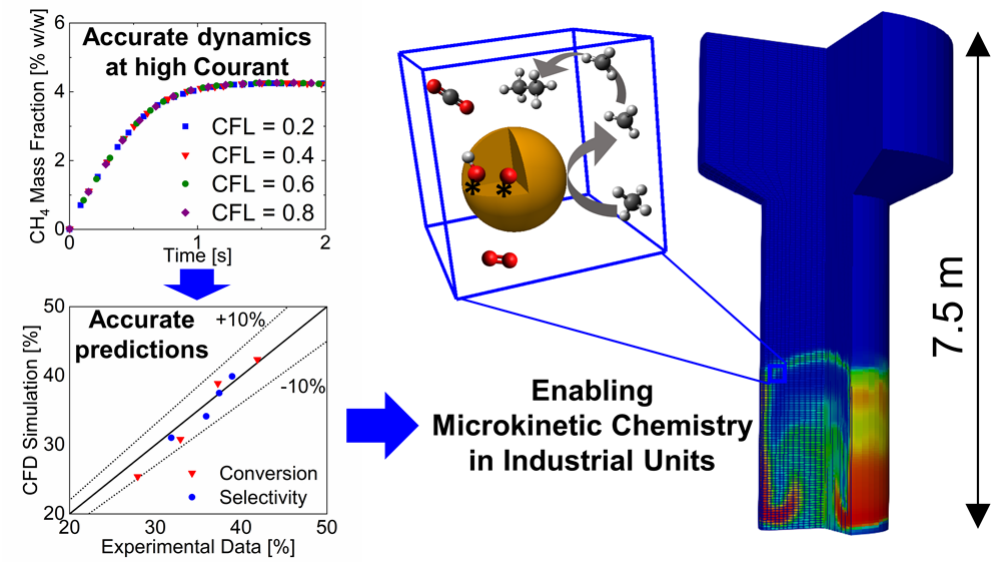

We propose a numerical methodology to combine detailed microkinetic modeling and Eulerian–Eulerian methods for the simulation of industrial fluidized bed reactors. An operator splitting-based approach has been applied to solve the detailed kinetics coupled with the solution of multiphase gas–solid flows. Lab and industrial reactor configurations are simulated to assess the capability and the accuracy of the method by using the oxidative coupling of methane as a showcase. A good agreement with lab-scale experimental data (deviations below 10%) is obtained. Moreover, in this specific case, the proposed framework provides a 4-fold reduction of the computational cost required to reach the steady-state when compared to the approach of linearizing the chemical source term. As a whole, the work paves the way to the incorporation of detailed kinetics in the simulation of industrial fluidized reactors.

## Introduction

Catalytic gas–solid fluidized reactors are of great interest in the chemical and energy industry. In particular, these systems allow for the operation of challenging processes in the context of the fuels synthesis (e.g., Fischer–Tropsch,^1,2^ biomass gasification^3,4^), fuel upgrading (FCC^5−8^), and anhydrides production.^9,10^ Moreover, they represent a promising reactor configuration for novel green and sustainable processes, e.g., chemical looping combustion^11,12^ for CO_2_ capture, methane conversion to nanostructured carbon materials and hydrogen,^13,14^ and oxidative coupling of methane −OCM–^15,16^ for natural gas valorization.

In this context, multiscale modeling^17,18^ has been acknowledged as a promising route to analyze such systems in order to assist with the experimental investigations^19,20^ for design and scale-up purposes. The macroscopic behavior of the reactive fluidized system is the result of the coupling between different time and length scales. In particular, the observed reactivity of the system is governed by the kinetics of the elementary steps at the atomistic scale under the conditions of chemical potential at the catalyst surface. At the same time, the species distribution inside the unit is determined by their transport in the reactor and hence by the fluid dynamics at the reactor scale, which is strongly related to the movement of the solid phase. Thus, the description of the gas–solid multiphase flow inside the reactor and its complex interplay with the chemical phenomena are pivotal to the detailed analysis of these systems.^21^

The multiscale modeling approach has been applied by Maestri and co-workers to lab-scale fluidized beds^20,22^ by extending the methodology developed for fixed bed reactors.^23−25^ In doing so, the fluid behavior and the transport phenomena in the reactor are described through the solution of the governing equations using computational fluid dynamics (CFD).^23^ At the same time, the accurate description of the gas-phase and the heterogeneous catalytic kinetics is obtained by means of first-principles kinetic model.^23^ In particular, the Euler–Lagrange^26−28^ CFD modeling approach, based on individual particle tracking, has been combined with the microkinetic description of the catalytic chemistry to investigate such systems. This approach is, however, not suitable for describing industrial units (i.e., target of this work). In fact, the CFD investigation of relevant units is usually performed in the literature by selecting a Eulerian–Eulerian description of the gas–solid multiphase flow. According to this approach, the simulation of industrial units is allowed by substituting the tracking of each solid particle inside the reactor with a Eulerian description of the solid phase. However, this numerical approach is an open research field for the improvement of both fluid dynamic and chemistry predictions. From the fluid dynamic standpoint, the Eulerian–Eulerian description of the multiphase flow has to be refined by considering the effects of particle clustering. In particular, these structures must be properly accounted for because they affect the fluid dynamic of the system in both laminar and turbulent regimes. On the one hand, proper models are needed if a computational domain with a grid having a size larger than the cluster of particles one is adopted to divide the computational domain.^29^ In fact, this computational grid is not able to describe the effect of the particle clustering, resulting in an inaccurate description of the multiphase flow. On the other hand, the particle clusters affect also the turbulent regimes,^30^ and their effects have to be properly accounted for in the Euler–Euler simulations. Nevertheless, the Euler–Euler modeling approach has been frequently adopted in the literature to describe the fluid-dynamic behavior of industrial systems (i.e., bubbling beds,^31,32^ turbulent fluidized bed^33^ of about 3 m, circulating fluidized bed^34^ of about 10 m, FCC riser^35^ of about 15 m), with good results.

From the chemistry standpoint, in the Euler–Euler literature, the introduction of chemistry has been performed by adopting rate equation kinetics.^36−39^ This assumption has been made in order to avoid the numerical coupling between the long reactor dynamics, characteristic of industrial units, and the fast elementary steps. However, it causes a loss of information about gas radicals and adsorbed species,^40^ fundamental for the investigation of the chemical phenomena. In addition, the reactor scale species transport and the chemistry of the macrospecies are usually solved coupled by performing the linearization of the rate equation in the simulation time step, leading to the use of the same temporal discretization for both the kinetic and fluid-dynamic phenomena. The linearization procedure intrinsically requires short time steps (i.e., 10^–5^ to 10^–4^ s) due to the strong nonlinearity and stiffness of the reaction source terms. For this reason, the Courant–Friedrichs–Lewy condition (CFL) adopted in the literature simulations is usually limited (i.e., 10^–2^ to 10^–1^) in order to minimize the linearization errors.^36−38^ A first attempt to overcome this limitation has been recently proposed by Vandewalle et al.^41^ They substitute the linearized source terms with constant production and consumption rates over the time step. According to their solution algorithm, these constant rates are evaluated by averaging the temporal trends of the reactive source term obtained by solving the sole chemistry with an ODE solver. The methodology enables an increase of the CFL, reducing the limitations on the time step of the linearized approach. However, the simulation results are still a function of the selected time step since it affects the averaging of the rates.^41^

A numerical strategy able to overcome these issues has been proposed in the literature for the CFD description of reactive fixed beds^23^ and lab-scale fluidized beds.^20^ The numerical coupling of the transport phenomena and the chemical reactions is efficiently carried out by means of the Strang operator-splitting algorithm.^42,43^ In doing so, the species transport and reaction operators are solved sequentially during a simulation time step, by managing the numerical stiffness of the chemistry by an ODE solver. This allows avoidance of the linearization of the reactive source term and performance of a short substep only for the computational cell-wise solution of the chemistry. This approach was demonstrated to be effective in coupling CFD with the microkinetic description of the chemistry with mean-field or kinetic Monte Carlo models, as shown by Maestri and co-workers.^20,22−24,44^

In this work, we extend the operator splitting to the CFD Euler–Euler modeling of fluidized bed systems coupled with detailed microkinetic modeling of the gas-phase and heterogeneous kinetics. In particular, we propose the multiphase operator-splitting (MOS) algorithm. First, the multiphase Navier-Stokes equations are solved to track the gas and solid fluid-dynamic behavior. Then, the solution of the species advection is computed. Finally, each computational cell is integrated as a multiphase batch reactor which accounts for the gas-phase reactions, the heterogeneous reactions and the gas–solid interphase transport.

The MOS Euler–Euler methodology has been first compared with the literature linearized chemistry approach in a lab-scale fluidized bed reactor,^40^ by means of a literature microkinetic mechanism^45−47^ of the OCM process. Then, the accuracy of the MOS Euler–Euler approach has been assessed by comparing the obtained prediction of the outlet reactor composition with the OCM experimental results collected by Jašo et al.,^40^ leading to a maximum deviation of less than 10% for methane conversion and 5% for C_2_ selectivity. Finally, the applicability of the methodology to industrial interest units is shown applying the same microkinetic mechanism. As a whole, this work paves the way for the application of a reactive multiscale modeling to industrial scale fluidized units, representing a tool for the design and scale-up of novel fluidized technology.^48,49^

## Numerical Method

### Governing Equations

The continuity equations for the gas and the solid phase are reported in eqs 1 and 2, respectively:
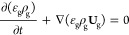
1
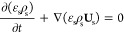
2where the subscripts g and s represent the gas and the solid phases, ε_g_ and ε_s_ are the gas and solid volume fractions, defined as the ratio between the volume occupied by the gas or the solid and the total volume of the cell, ρ_g_ and ρ_s_ are the gas and solid densities, **U**_**g**_ is the gas velocity vector, and **U**_**s**_ is the average solid phase velocity.

The momentum equations for the gas and the solid phase are reported in eqs 3 and 4:
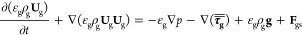
3
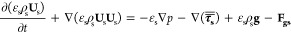
4where **F_gs_** refers to the gas–solid momentum transfer, *p* is the pressure of the system, **τ̿**_**g**_ is the gas stress tensor, and **τ̿**_**s**_ is the solid stress tensor derived from the assumption of a continuum solid phase.

The gas stress tensor **τ̿**_**g**_ has been modeled by means of the Newton stress tensor. The continuum property of the solid phase (i.e., **τ̿**_**s**_) is obtained by means of the kinetic theory of granular flow (KTGF)^50^ as a function of the granular temperature θ:
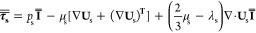
5
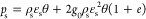
6
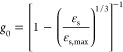
7where *p*_s_ is the solid pressure (eq 6), *e* is the restitution coefficient, μ_s_ and λ_s_ represent the solid shear viscosity and the solid bulk viscosity, modeled in this work as a function of the granular temperature θ. The radial solid distribution (*g*_0_) depends on the solid volume fraction, and it is modeled according to the correlation proposed by Sinclair and Jackson^51^ as reported in eq 7, where ε_s,max_ represents the random close-packing limit solid volume fraction, i.e., 0.64.

According to the KTGF, the granular temperature θ, defined in eq 8, represents the statistical behavior of the solid phase by taking into account all the information losses with the averaging procedure.
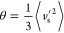
8where *v*_s_^′^ is the fluctuation of the particle velocity with respect to the volume average solid phase velocity **U**_s_, and the bracket represents the average of the fluctuation of all the particles present within a finite volume. The temporal evolution of the granular temperature θ is described by means of eq 9:
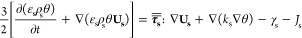
9where *k*_s_ is the solid conductivity, γ_s_ represents the dissipation of granular energy related to the inelastic particle–particle collisions, and *J*_s_ represents the dissipation of granular energy related to the momentum exchange between the two phases. This equation has been formulated by accounting for the terms of the solid phase momentum balance equation, related to the velocity fluctuations, lost during the averaging procedure necessary to obtain eq 4. In the Euler–Euler framework, closure models are needed to describe *k*_s_, γ_s_, and *J*_s_. The closure models usually adopted in the literature are used in this work and listed in section 1 of the Supporting Information.

The species mass balances for the gas and the solid phases are reported in eqs 10 and 11, respectively:
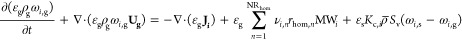
10
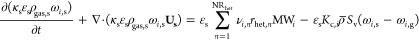
11where ω_*i*_, MW_*i*_, and *K*_*c*,*i*_ represent the *i*th species’ mass fraction, molecular weight, and mass transfer coefficient; ρ_gas,s_ is the density of the gas inside the solid phase, ρ̅ is the average density between ρ_g_ and ρ_gas,s_; *S*_v_ is the surface to volume ratio of the catalytic particles; **J**_***i***_ is the diffusive flux of the *i*th species; and κ_s_ is the porosity of catalyst particles. The subscripts hom and het represent the homogeneous and heterogeneous reactions, respectively. NR is the number of reactions. *r*_*n*_ is the *n*th reaction rate, and ν_*i*,*n*_ is the *i*th species stoichiometric coefficient in the *n*th reaction.

The equation describing the evolution of the site fraction of the *j*th adsorbed species must be included to allow for the microkinetic description of the heterogeneous chemistry:
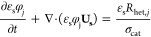
12where φ_*j*_ and *R*_het,*j*_ are the coverage and the production rate due to the heterogeneous reactions of the *j*th adsorbed species and σ_cat_ is the concentration of the active site on the catalytic surface. In particular, differently from the multiscale modeling approach of catalytic fixed bed reactors,^24^ the convection contribution is added to eq 12 since the adsorbed species are transported between the computational cells due to the movement of the solid phase.

The energy equations for the gas and the solid phase are reported in eqs 13 and 14, respectively:
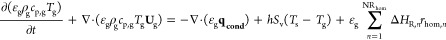
13
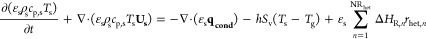
14where *T* is the temperature, *c*_p_ the specific heat capacity, **q**_**cond**_ is the conduction heat flux, Δ*H*_R,*n*_ represents the reaction enthalpy of the *n*th reaction, and *h* is the heat transfer coefficient. The gas mixture is assumed as an ideal mixture of ideal gas. The internal heat and mass transfer limitation have been neglected due to the fine size of the investigated particles, i.e., on the order of 10^–4^ m. Thus, a uniform temperature and composition distribution has been assumed in the solid phase. The energy dissipation due to the viscosity of the fluid is neglected, and the pressure term is ignored.^52^ Different closure models are present in the literature to describe the heat and mass transfer, the convective and diffusive fluxes and the gas–solid drag force. The ones adopted in this work are reported in section 1 of the Supporting Information. In particular, the correlation proposed by Gunn^53^ has been employed to describe the heat and mass transfer coefficient because it accounts for the void fraction of the computational cells.

The transport properties and the homogeneous reactions are evaluated by means of the OpenSMOKE++ libraries,^54^ while the heterogeneous reactions are evaluated by means of the catalyticSMOKE libraries present in the catalyticFOAM framework.^23^ The volume averaged continuity, Navier–Stokes, energy, and species equations are discretized and solved according to the finite volume method implemented in the OpenFOAM^55^ framework.

### Multiphase Operator Splitting

**Figure 1:**
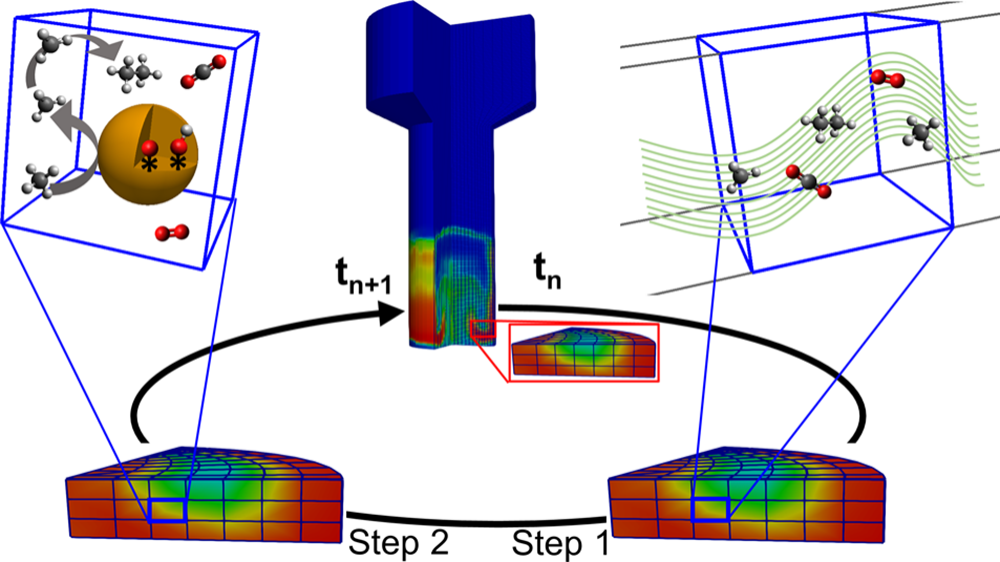
Schematic representation of the multiphase operator splitting (MOS) approach. In step 1, the chemical species and the energy are transported among the computational cells, while in step 2 the chemical phenomena happen sequentially to the transport step.

In this work, multiphase operator splitting (MOS) is proposed to tackle the solution of the chemistry in the reactive Euler–Euler methodology. This numerical approach is derived by adapting the operator splitting,^42,43^ developed to treat gas-phase chemistry, to multiphase reactive flows. The MOS approach divides the solution of a simulation time step into two substeps, as shown in Figure 1.

In the first substep (Figure 1), the species and energy balances are solved by only considering the convection and diffusion transport over the time step, as follows:
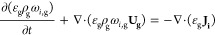
15
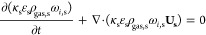
16
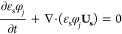
17

All the equations of this step are solved sequentially, according to the segregated approach. In particular, their discretized form generates an algebraic linear system of equations due to the negligible nonlinearity of the transport phenomena. Thus, an iterative matrix solver has been adopted to compute the evolution of this substep. The obtained solution updates the composition and temperature of each computational cell for both the gas and solid phases. This condition is then set as an initial condition for the second substep (Figure 1). In the second substep, each computational cell becomes a multiphase batch reactor described by an ODE system derived by only considering the homogeneous chemistry, the gas–solid mass transfer, and the heterogeneous chemistry contributions in the gas (eq 18) and the solid (eqs 19 and 20) phase governing equations:
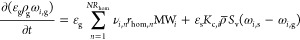
18
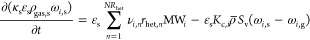
19
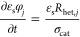
20

The equations of the second substep are solved coupled by means of an ODE solver, able to account for the nonlinearity of the chemistry source term. The sequence of the steps performed in the MOS approach has been selected in order to follow the splitting procedure adopted in the operator splitting literature.^56^ Indeed, the stability of a splitting algorithm is related to the sequence of the splitting, as discussed by MacNamara and Strang.^57^ In particular, in this work we have tested both the configurations (transport step followed by reaction step and reaction step followed by transport step), but the second one is less robust than the one adopted in the MOS.

In order to minimize the error related to the splitting of the phenomena, the simulation time step (i.e., the splitting time) must be chosen according to the characteristic time of the involved transport and chemical phenomena. In particular, a reactive fluidized system is characterized by phenomena (i.e., species advection, gas–solid interphase transport and chemistry) having a wide range of characteristic times. The characteristic time of the species advection is related to the time necessary to pass through a cell of the computational domain (i.e., 10^0^ to 10^–2^ s). The specific interphase area of the catalytic particles adopted in fluidized processes (i.e., 10^4^ to 10^5^ m^–1^) leads to a small characteristic time of the interphase transport (i.e., 10^–5^ to 10^–7^ s). Finally, the chemical events usually have a characteristic time in the range of 10^–6^ to 10^–10^ s. By applying the MOS approach, the time substeps performed by the ODE solver to describe the species evolution in the computational cell account for the stiffness and the characteristic time of the fast phenomena present inside the system (i.e., chemical kinetics and interphase transport). At the same time, the CFL allows the correct description of species advection. Therefore, the proposed approach minimizes the errors introduced by the numerical strategy adopted to describe the detailed chemistry. In doing so, the adoption of an ODE solver in the second step of the MOS approach allows selection of the optimal substep according to the fastest phenomena present in the system allowing the simulation of both systems characterized by an interphase diffusion faster than the chemical events (i.e., chemical regime) or by chemical events faster than the interphase transport (i.e., mass transfer regime), as shown in section 2 of the Supporting Information. Moreover, the ODE solver increases the flexibility of the methodology. Consequently, the second step of the proposed approach can be modified in order to include phenomena neglected in this work (i.e., intraparticle limitations).

For these features, the proposed approach allows for employing a CFL higher than the one typically used in the literature reactive Euler–Euler simulations (i.e., 10^–2^ to 10^–1^),^36−38^ thus it is able to overcome the limitation related to the linearization of the chemistry source term. However, the optimal CFL has to be chosen for each process under investigation.

## Simulation Setup

The proposed framework has been tested by considering the oxidative coupling of methane (OCM) on the La_2_O_3_/CaO catalyst^58^ as an example in a lab-scale and an industrial scale reactor. The kinetic mechanism selected to include the OCM chemistry in the Euler–Euler framework, the computational domain, and the boundary conditions employed are described in this section.

### OCM Reaction Kinetics

The OCM involves both homogeneous and heterogeneous chemistries. In this work, we combine two microkinetic schemes for their detailed description. With respect to the heterogeneous chemistry, it is described by means of the microkinetic scheme proposed by Simon et al.,^45^ composed of 21 reactions involving five adsorbed species. With respect to the homogeneous chemistry, the microkinetic scheme accounting for main gas-phase reactions that occur in the OCM process selected by Sun et al.,^46^ composed of 39 reactions involving 24 gaseous species, has been adopted. This microkinetic scheme does not include the evolution of C1 radicals, necessary for the heterogeneous schemes. Consequently, these reactions are accounted for by means of the scheme proposed by Zanthoff et al.,^47^ composed of nine reactions involving 13 gaseous species. All of the reactions and the kinetic parameters are reported in section 3 of the Supporting Information.

### Computational Domains

**Figure 2:**
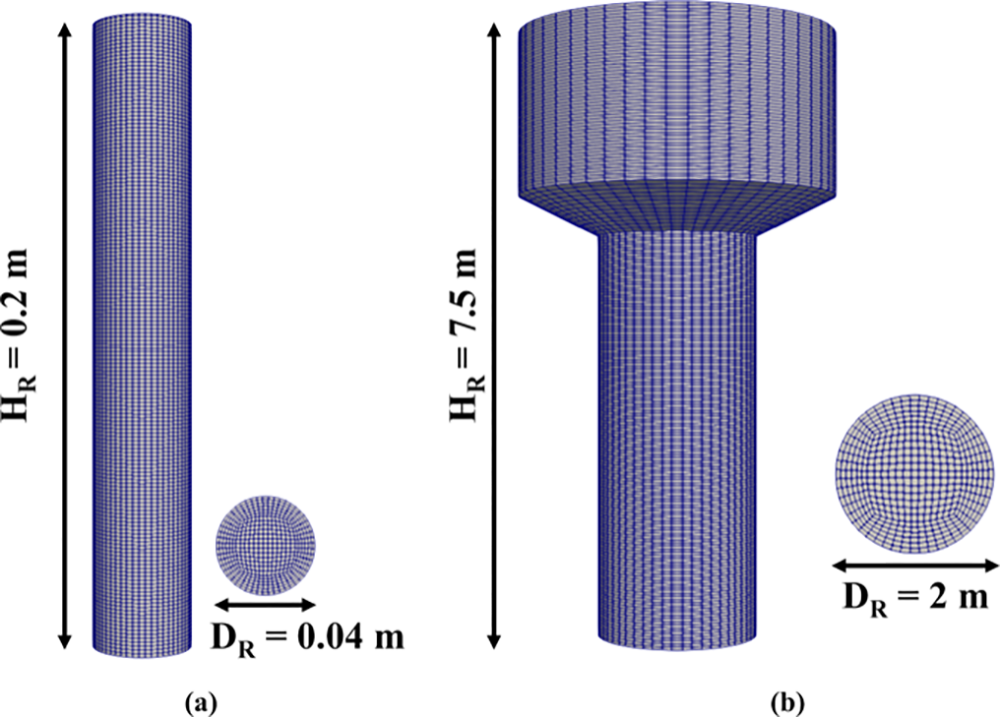
Computational domain of the lab-scale reactor configuration (a) and the industrial-scale reactor configuration (b).

Two 3D computational domains, shown in Figure 2, have been selected for the simulations reported in this work.

The first computational domain has been generated to reproduce the lab-scale reactor experimentally investigated by Jašo et al.^40^ It is a cylindrical reactor consisting of a 4 cm diameter tube, as shown in Figure 2a. The dimension of the cubic cells composing the computational grid has been selected to be equal to 10 times the average particle diameter, leading to 63,973 computational cells having an average volume of 3 × 10^–9^ m^3^.

The second computational domain has been generated to reproduce a conventional industrial fluidized bed reactor, shown in Figure 2b. It is composed of a cylindrical section of 2 m diameter, followed by an enlargement up to 4 m to disengage the particles entrained by the gas flow. In this reactor, the dimension of the cubic cell has been selected to be equal to 500 times the average particle diameter, leading to 95,719 computational cells having an average volume of 4.64 × 10^–4^ m^3^. The geometrical and mechanical properties of both the reactor and the particles are specified in Table 1. The mesh convergence analysis for the two computational domains is reported in sections 4 and 5 of the Supporting Information.

**Table 1:** Geometrical and Mechanical Properties of the Two Reactor Configurations and the Catalytic Particles

	lab scale	industrial scale
reactor diameter, *D*_R_ [m]	0.04	2
reactor height, *H*_R_ [m]	0.2	7.5
initial bed height, *H*_0_ [m]	0.04	1
computational cells, *N*_cell_ [-]	63,973	95,719
cell to particle ratio, Δ*x*/*d*_p_ [-]	10	500
particle diameter, *d*_p_ [μm]	150	150
particle density, ρ_s_ [kg/m^3^]	3600	3600
restitution coefficient, *e* [-]	0.9	0.9

The initial condition of the fluidized bed simulations is a packed bed whose height is user-defined by the loaded mass of the catalyst and the packed bed void fraction. The initial solid fraction has been set equal to 0.62, due to the high reactor to particle ratio (*D*/*d*_p_ > 270). Consequently, the initial bed height has been set equal to 4 cm for the lab-scale reactor and to 1 m for the industrial-scale one, computed from a catalyst loading of 0.112 kg and 7.012 ton, respectively. The packed beds are initially fluidized by using an inert flow of nitrogen injected from the bottom of the reactor. Once a steady fluidization is reached, the reactive feed, composed of methane, oxygen, and nitrogen, is injected into the reactor from the bottom. The systems have been simulated under isothermal conditions. This assumption is consistent with the experimental setup since a maximum hotspot lower than 10 °C has been observed in the lab-scale reactor.^59^ We have also considered that this hypothesis is valid in the industrial scale configuration due to its fluidization ratio (i.e., 45), which guarantees high mixing and a homogeneous reactor environment.

As boundary conditions, atmospheric pressure has been fixed at the top of the reactor, while a zero-gradient condition has been assumed for the lateral walls and for the bottom. The superficial velocity has been imposed at the bottom of the reactor equal to 5 times the minimum fluidization velocity for the lab-scale configuration (leading to a Reynolds number, *Re*_p_, equal to 0.102) and to 45 times the minimum fluidization velocity for the industrial scale configuration (leading to a *Re*_p_ = 0.92). According to the fluid velocity, different fluid regimes can appear in the fluidized bed reactor from laminar to fully turbulent flow. Due to the complex description of the effects of the turbulence in gas-particle flows,^30^ we performed an assessment of the flow regime present inside the industrial units according to several correlations reported in the review of Bi et al.^60^ The turbulent phenomena start becoming important inside a fluidized environment after a critical Reynolds number that depends on the fluid and particles properties. We computed this parameter for the selected configuration obtaining a critical Reynold value in the range 2.57–5.4 significantly above the *Re*_p_ considered in this work. Consequently, we neglect the presence of turbulent phenomena also in the industrial configuration. At the lateral walls, a no-slip boundary condition has been set for the gas-phase velocity. For the solid phase velocity, the boundary condition proposed by Johnson and Jackson^61^ has been imposed at the bottom of the reactor and at the lateral walls. The gas-phase composition has been imposed at the bottom of the reactor according to the operating conditions of the inlet feed stream. A Neumann condition has been imposed on the remaining boundaries for the species and temperature. Moreover, a specific surface area of the catalyst must be set in order to use the heterogeneous microkinetic scheme. This quantity has been set equal to 1.4487 × 10^5^ m_cat_^2^/m_cat_^3^ (computed by considering an average diameter of the clusters of active sites equal to 10 nm) in all the simulations performed in this work. We have assessed its influence on the simulation results by parametrically changing the value in the range ±10%. We observed deviations lower than 2.5% in the methane conversion, as discussed in section 6 of the Supporting Information.

## Results and Discussion

The hereby proposed Euler–Euler multiscale framework was first numerically assessed in the lab-scale reactor configuration comparing its prediction with the one obtained by linearizing the reactive source term. Then, we present two showcases. The first showcase is a lab-scale reactor experimentally investigated by Jašo et al.^40^ adopted to validate the outcome of the proposed framework with experimental data. Finally, the second showcase proves the applicability of the validated framework in an industrial scale reactor configuration. In all of the tests of the Euler–Euler MOS, the oxidative coupling of methane has been selected only as an example process, thus, OCM reactor design purposes are out of the scope of this work.

### Assessment of the Multiphase Operator Splitting (MOS)

The MOS algorithm has been tested simulating the lab-scale configuration in a range of CFLs of 0.2–0.8 to assess their effect on the simulation. Moreover, the same simulations have been performed also by means of the linearization of the chemistry source term typically employed in the reactive Euler–Euler literature approach. The operating conditions adopted for the numerical tests are a reactor temperature equal to 1023.15 K and a feed composition consisting of 0.1 v/v of methane, 0.04 v/v of oxygen, and 0.86 v/v of nitrogen. Methane (main reactant of the OCM process) has been considered as a reference species for comparing the predictions of the two algorithms, for all the tested CFLs. The numerical assessment was performed by considering the methane mass fraction averaged over the whole fluidized bed, calculated as in eq 21:
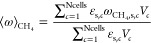
21where Ncells is the number of cells in the computational domain and *V*_*c*_ is the volume of the *c*th computational cell.

In doing so, not only the prediction of the methane fraction leaving the reactor but also the map of methane inside the catalytic bed are accounted for in a macroscopic parameter.

**Figure 3:**
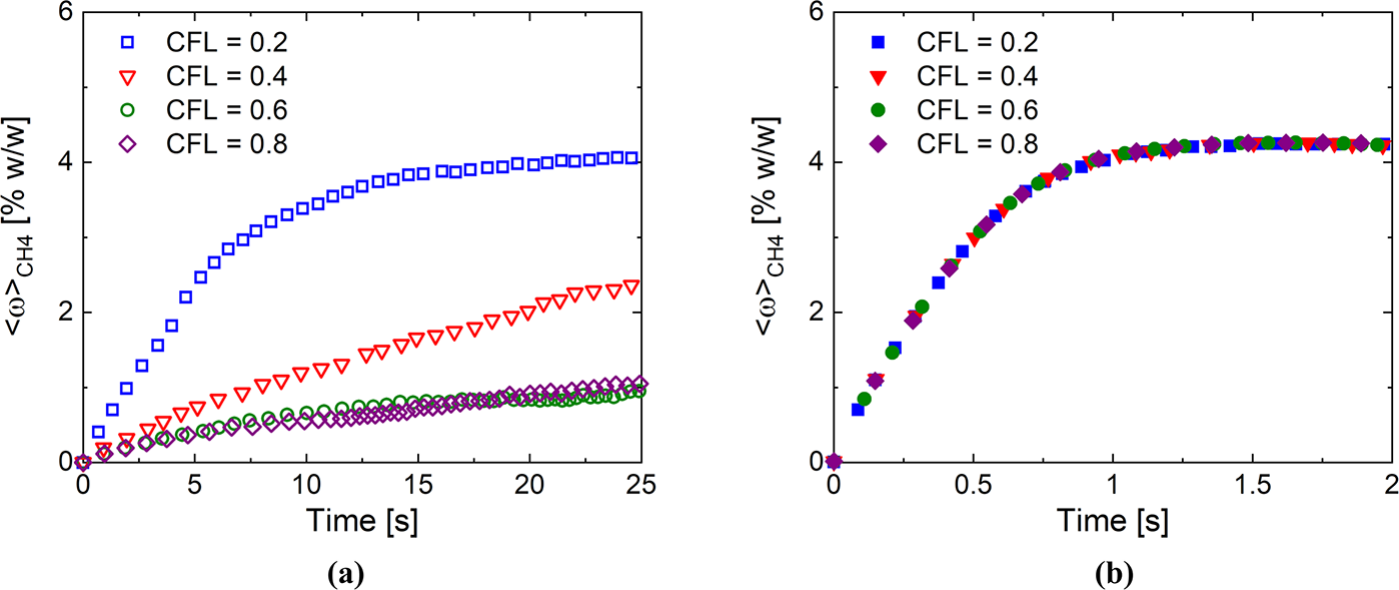
Temporal trend of the volume average methane mass fraction in the catalytic bed obtained with the linearization of the reactive source term over 25 real seconds (a) and the MOS approach over 2 real seconds (b) at different CFLs.

Figure 3 shows the trend of the volume averaged methane mass fraction ⟨ω⟩_CH_4__ in the catalytic bed as a function of the time. The dynamic of the simulated system obtained with the linearized Euler–Euler approach is strongly dependent on the CFL, as shown in Figure 3a. Indeed, the CFLs used to perform this assessment are an order of magnitude higher than the one usually used in the literature (i.e., 10^–2^) to minimize the linearization errors. In contrast, the hereby proposed framework shows a negligible dynamic influence with respect to the CFL, as shown in Figure 3b. Indeed, the discrepancies between the four investigated CFLs simulated with the MOS-based Euler–Euler framework lead to a maximum difference up to 0.5% during the dynamic evolution of the system. Thus, the splitting procedure adopted in the MOS approach is able to respect the characteristic time of all the phenomena occurring in the system. This leads to the absence of the time step limitations required by the linearized approach, being the deviation of the MOS minimized thanks to the time substepping of the ODE solver in case of chemistry/interphase transport coupling.

**Figure 4:**
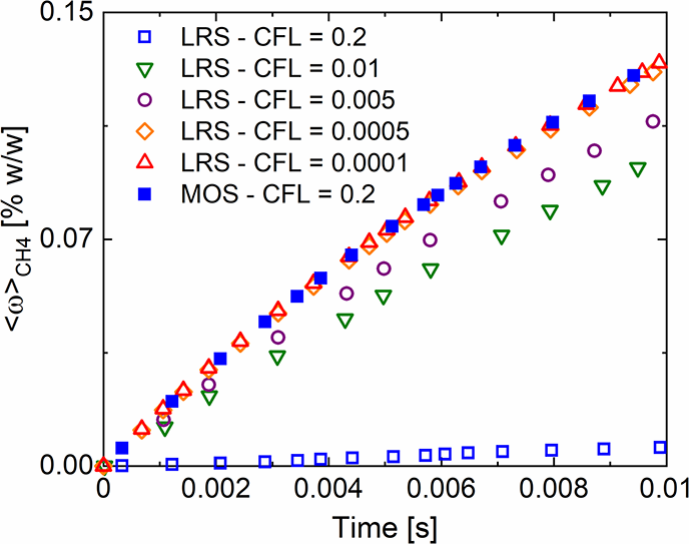
Temporal trend of the volume average methane mass fraction in the catalytic bed obtained with the linearization of the reactive source term (LRS) at different CFLs (open symbols) and with the MOS approach at a CFL equal to 0.2 (blue squares).

Consequently, we adopt the MOS solution as a benchmark to test the prediction of the linearized reactive Euler–Euler approach. In particular, we simulated the linearized reactive Euler–Euler approach by considering the same reactor and operating conditions by means of a wider CFL range, i.e. 10^–4^-0.2, coherent with the CFL employed in literature.^36−38^Figure 4 shows the trends of the volume averaged methane mass fraction as a function of time for the different CFLs investigated with the linearized approach. Only the first 0.01 s of simulation have been performed due to the high computational cost required by the lower CFL. It is evident that by significantly decreasing the CFL, the predictions of the linearized Euler–Euler are in accordance with the one obtained with the MOS approach. In particular, a CFL smaller than 5 × 10^–4^, leading to an average time step equal to 3 × 10^–8^ s, is necessary to have a dynamic that is not influenced by the time step with the linearized framework. Consequently, the linearized reactive Euler–Euler modeling approach results not applicable for managing the dynamics of the fluidized units by adopting a detailed chemistry description, as reported by Jašo et al.^40^

Therefore, the MOS algorithm allows for the simulation of the reactor with a 4.3-fold reduction of the computational time required to achieve the pseudo-steady-state condition with respect to the linearized approach. Indeed, this speed-up is the result of a 20.8-fold reduction of the total number of simulation time steps (at CFL = 0.2) which compensates for the 4.8-fold slow-down introduced by the ODE solver.

In summary, this analysis shows that the linearization of the source term may introduce in this case a nonrealistic delay in the dynamic evolution of the reaction environment, thus requiring a significantly higher number of time steps compared to the MOS approach to achieve the pseudo-steady-state of the system which is however not affected by the chosen numerical approach, as expected from the literature results obtained with linearized Euler–Euler and shown in Figure 3. The numerical explanation of the delay obtained with the linearized approach is reported in section 7 of the Supporting Information. Next, we consider two different reactor configurations: the first one aimed to validate the prediction of the MOS-based Euler–Euler approach with experimental data, and the second one aimed to show the applicability of the framework to industrial systems.

### Showcase 1

The fluid-dynamic behavior and the reactive predictions obtained with the MOS-based Euler–Euler framework have been assessed by means of a comparison with the theoretical pressure drop and experimental conversion and selectivity values, respectively.

**Figure 5:**
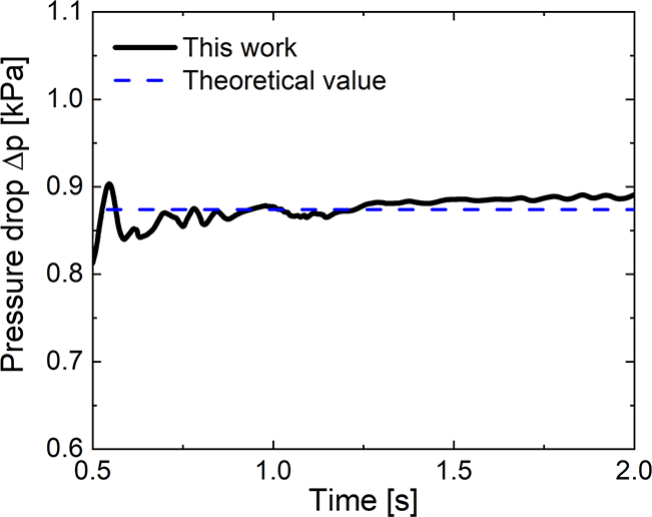
Lab-scale configuration: temporal trend of the pressure drops (solid line) compared with the theoretical value (dashed line) equal to 874.3 Pa.

Figure 5 shows the temporal profile of the pressure drop computed with the proposed Euler–Euler approach as the difference between the pressure at the top and at the bottom of the computational domain. This profile is characterized by an oscillating behavior caused by the continuous expansions and contractions typical of fluidized beds. The temporal average has been compared with the theoretical value (i.e., the ratio between the weight of the bed and the cross-sectional area of the reactor) leading to an excellent agreement with a deviation of around 1.2%.

The chemical outcomes of the MOS-based Euler–Euler framework are assessed by means of the experimental data collected by Jašo et al.^40^ in the same reactor geometry. In particular, the methane conversion and the selectivity to the C_2_ compounds (i.e., ethylene, ethane, and acetylene) predicted by the MOS-based framework have been evaluated by means of the cup-mixing average of the species mass fractions. The averaging plane has been set at a height of 0.07 m, just above the maximum expansion of the fluidized bed, in order to neglect the freeboard, coherently with the procedure employed in the experimental campaign.^40^

Different operating conditions have been considered to compare the chemistry predictions by changing the oxygen fraction of the feed, as reported in Table 2, following the experimental campaign of Jašo.^40^

**Table 2:** OCM Operating Conditions Adopted to Assess the MOS-Based Euler–Euler Framework

operating conditions					
			inlet molar fraction [v/v]		
case	temperature [K]	pressure [Pa]	CH_4_	O_2_	N_2_
A	1023.15	10^5^	0.1	0.02	0.88
B	1023.15	10^5^	0.1	0.03	0.87
C	1023.15	10^5^	0.1	0.04	0.86
D	1023.15	10^5^	0.1	0.05	0.85

**Figure 6:**
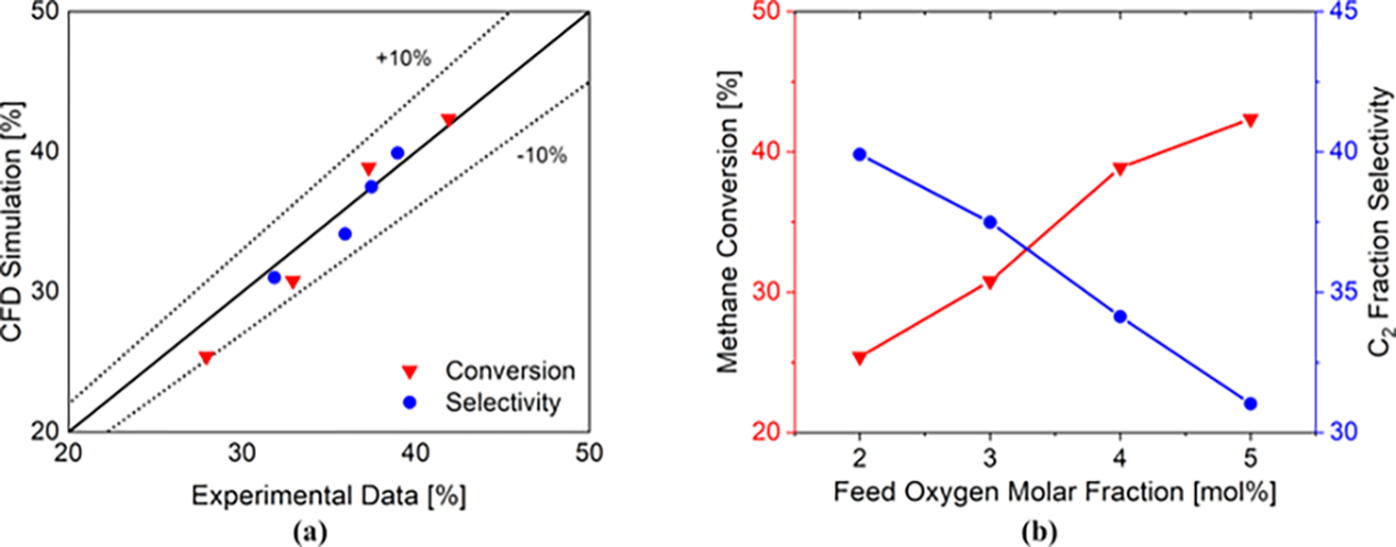
Lab-scale configuration: (a) parity plot comparing the numerical results obtained with the MOS-based Euler–Euler proposed in this work with the experimental data collected by Jašo et al.^40^ in terms of methane conversion (red triangles) and selectivity to the C2 fraction (blue circles); (b) trend of the methane conversion (red line with closed triangles) and C2 fraction selectivity (blue line with closed circles) at the outlet of the catalytic bed (0.07 m height) predicted by the MOS-based Euler–Euler.

The comparison of the Euler–Euler results with the data experimentally collected is shown in the parity plots of Figure 6a.

The combination of the Euler–Euler framework with the detailed chemistry prediction is able to accurately predict the experimental data. Indeed, a good agreement is obtained in terms of both methane conversion and C_2_ fraction selectivity, since a maximum error up to 10% is obtained for the conversion, and up to 5% for the selectivity.

Figure 6b shows the methane conversion and the C_2_ fraction selectivity obtained with the MOS-based Euler–Euler framework for the investigated cases. The increment of the oxygen feed composition increases the overall reactivity of the system, leading to an increasing methane conversion. At the same time, it increases the rate of the reactions that produce the OCM side-products (i.e., CO_2_), leading to a decrement of the C_2_ fraction selectivity.

Thus, the combination of the MOS-based Euler–Euler framework with a detailed chemistry description is able to predict the experimental data.

### Showcase 2

Finally, the applicability of the MOS-based Euler–Euler framework to treat the detailed chemistry in units of industrial interest, related to the use of high CFL, is shown. To do so, an industrial scale configuration of the fluidized bed has been selected as the computational domain to show the potentiality of the numerical framework. The simulation has been performed by considering the OCM microkinetic mechanism as an example process, and a maximum CFL equal to 0.8 has been set, leading to an average time step of 2.43 × 10^–3^ s. The operating conditions adopted for this case are a reactor temperature equal to 1023.15 K and a feed composition consisting of 0.1 v/v of methane, 0.04 v/v of oxygen, and 0.86 v/v of nitrogen.

The fluid-dynamic predictions obtained with the Eulerian–Eulerian approach are strongly related to the closure model adopted to describe the gas–solid interactions, especially when a coarse grid (e.g., cell size larger than 100 d_p_) is used. In fact, the loss of the discrete nature of the solid particles leads to the absence of information regarding their distributions inside the computational cells. Consequently, the presence of complex fluid–solid structures (e.g., clusters of particles) can be lost if a coarse mesh is adopted, leading to inaccurate predictions of the fluid dynamic behavior of the system.^62^

The MOS-based Euler–Euler framework has been developed to employ different closure models to describe the gas–particle interactions. As discussed in the convergency analysis of the industrial scale configuration, reported in section 5 of the Supporting Information, the discretization of the computational domain by means of coarse grid (i.e., size larger than 100 d_p_) requires proper models to account for the loss of information related to the fluid–solid structures smaller than the computational cells. The overlook of these effects leads to an overestimation of the gas–particle interactions. However, this overestimation does not affect the fluidization regime, leading to negligible deviations on the chemistry predictions. Therefore, since the aim of this showcase is out of design purposes (i.e., shows the ability of the MOS approach to combine detailed microkinetic models with the long dynamics of industrial units), we select the correlation proposed by Gidaspow^50^ to describe the gas–particle interactions.

After 30 s of inert fluidization, the reactive mixture is injected into the computational domain and the simulation is performed until the pseudo-steady state. At each simulation time, the reactor outlet composition has been computed by means of the cup-mixing average at a height of 5 m.

**Figure 7:**
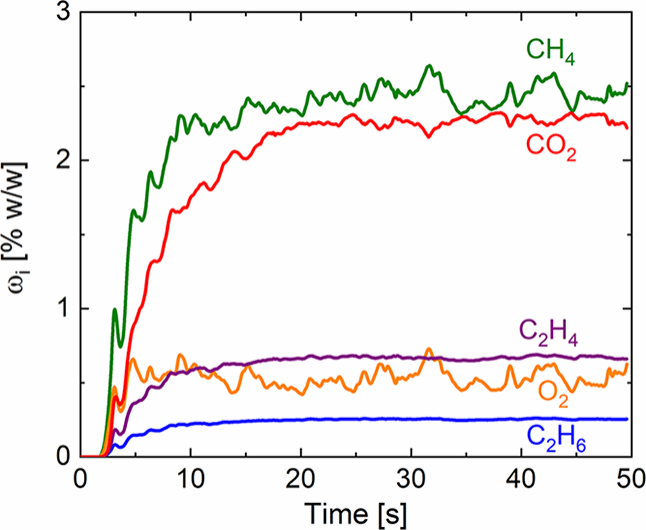
Industrial scale configuration: temporal trend of the cup-mixing mass fraction of methane (green), oxygen (orange), ethane (blue), ethylene (purple), and carbon dioxide (red).

Figure 7 shows the trend of the reactants (i.e., methane and oxygen) and the main reaction products (i.e., ethane and ethylene) and byproduct (i.e., carbon dioxide) mass fractions.

Similarly to the behavior of the pressure drops, the species mass fractions are also characterized by an oscillating trend related to the dynamics of the bubbles. In particular, the pseudo-steady state of this reactive system is reached after about three residence times, leading to a methane conversion of 58.6% and C_2_ selectivity of 32.1% obtained temporally averaging the species mass fraction over the last 20 s. In addition to the macroscopic chemistry performances of the system (i.e., conversion and selectivity), the proposed framework is able to provide information about the distribution of the macro-, radical, and adsorbed species inside the reactor.

**Figure 8:**
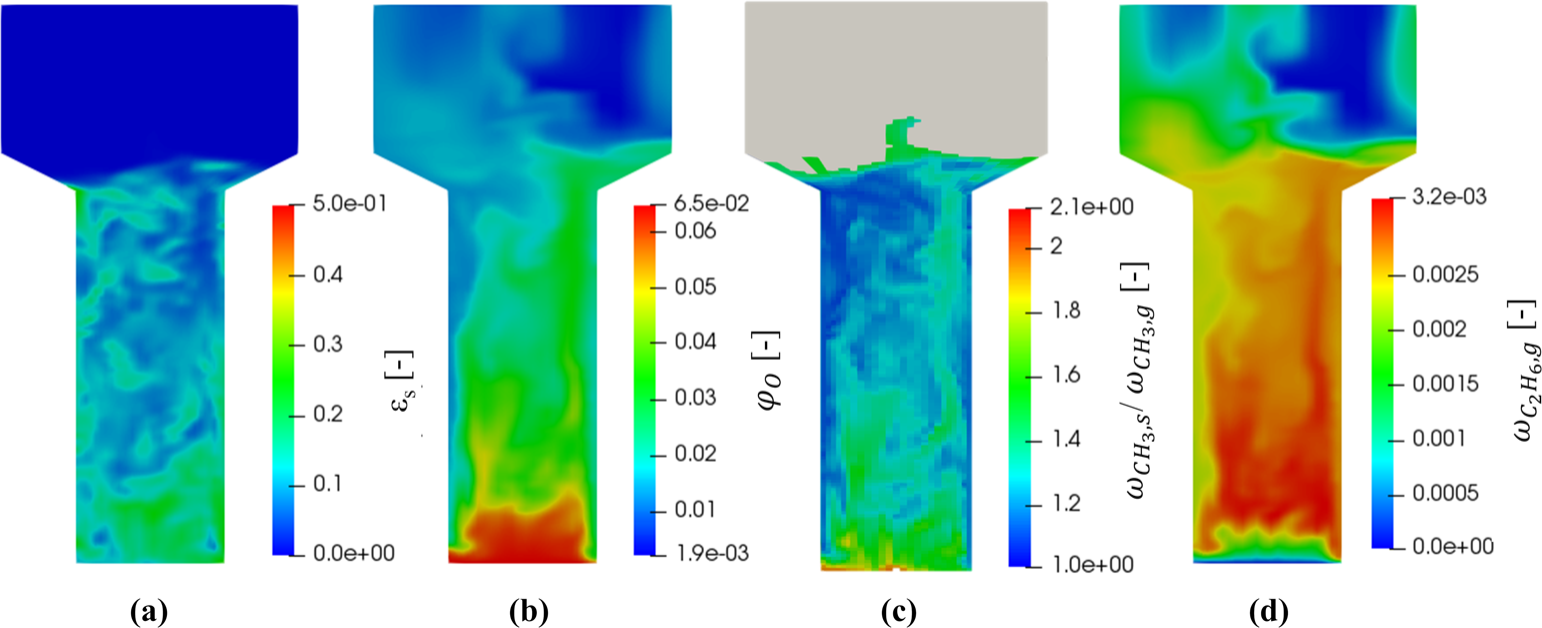
Industrial scale configuration: maps of the solid fraction (a), the adsorbed oxygen site fraction (b), the ratio between solid and gas-phase methyl radical mass fraction (computed in the reactor region characterized by a solid fraction higher than 10^–3^; c), and the ethane mass fraction (d) after 50 s of reactive fluidization.

Figure 8 shows the maps of the solid fraction, the adsorbed oxygen site fraction, the ratio between solid and gas-phase methyl radical mass fraction, and the ethane mass fraction obtained after 50 s of reactive simulation. The MOS-based Euler–Euler framework enables the assessment of the evolution of the species inside the system both temporally and spatially. Figure 8a shows that the reactor region close to the injection section is characterized by the highest amount of solid. Indeed, the mixing of the catalytic bed in this area is less effective due to the presence of the stagnation region close to the bottom reactor wall. Moreover, in this reactor zone, the catalytic particles are contacted by oxygen-rich flow, leading to a higher concentration of the adsorbed oxygen (Figure 8b). This species is responsible for the heterogeneous activation of the methane and for the consequent generation of the methyl radical. This radical is characterized by a high reactivity and, consequently, it is usually affected by mass transfer limitations in the OCM process. The aforementioned limitations cause the formation of gas–solid methyl radical gradients which are shown in Figure 8c by means of the methyl solid to gas mass fraction ratio. In particular, the regions characterized by a ratio higher than 1 are affected by the presence of the transport limitation, whereas the regions showing a ratio close to 1 are weakly influenced by interphase transport resistances. Finally, Figure 8d shows the spatial distribution of the ethane mass fraction, which is the result of methyl radical gas-phase combinations. The ethane, similarly to the other gas-phase species, is affected by the mixing behavior of the system. Indeed, an inhomogeneous ethane concentration is present in the reactor region characterized by a low mixing, while, a homogeneous distribution is present in the well-mixed zone.

Ultimately, the analysis of the computational cost has been performed. The industrial units are characterized by long dynamics, in the order of tens or hundreds of seconds (e.g., about 20 s in this case). In this work, the fluidization of the industrial scale configuration has been simulated for 30 s of real time in 1.6 h with 64 CPUs, leading to an average computational cost of 193.1 s per real second. At the same time, the MOS numerical approach allows for the adoption of the non-reactive CFL also for the reactive simulation. By doing so, an average computational cost of 5.9 h per simulated second is obtained for the reactive simulation of the industrial configuration by working with 64 CPUs and by considering the complex OCM microkinetic mechanism, composed of 48 homogeneous reactions and 21 heterogeneous reactions involving 24 gas-phase species and five adsorbed species.

## Conclusions

Microkinetic modeling has been coupled with the Euler–Euler framework by adopting the multiphase operator splitting (MOS) numerical approach. First, the proposed framework has been numerically tested and compared with the prediction of the linearized Euler–Euler approach in a lab-scale reactor configuration, by using a microkinetic mechanism describing the oxidative coupling of methane (OCM) process. The numerical test shows the capability of the solver to have a negligible influence on the CFL in the case under investigation. Indeed, contrary to the linearized Euler–Euler approach, a maximum deviation up to 0.5% during the dynamics is obtained by changing the maximum CFL from 0.2 to 0.8. By doing so, the MOS-based Euler–Euler approach is able to provide a 4.3-fold reduction of the computational cost required to reach the pseudo-steady state with respect to the linearized Euler–Euler approach.

Then, the fluid-dynamic and reactive predictions of the MOS-based Euler–Euler have been assessed. On the one hand, the temporal averaged pressure drop has been compared with the theoretical value leading to a deviation lower than 1.2%. On the other hand, the chemical outcomes have been compared with the experimental data collected by Jašo et al.^40^ leading to the correct description of the methane conversion and C_2_ fraction selectivity trends. Indeed, a deviation lower than 10% is obtained for the methane conversion and lower than 5% for the C_2_ fraction selectivity.

Finally, an industrial fluidized reactor configuration has been simulated by using the OCM microkinetic mechanism as example process in order to show the capability of the solver to treat relevant scale units. In particular, the MOS-based Euler–Euler approach is able to simulate the reactive system, obtaining both macroscopic design parameters (i.e., conversion, selectivity) and microscopic insights of the investigated processes (i.e., maps of the adsorbed species). To do so, an average computational cost equal to 5.9 h per real second with 64 CPUs is required to simulate the complex OCM microkinetic mechanism by setting a maximum CFL equal to 0.8, leading to the achievement of a pseudo-steady state condition in about 6 days.

## Supporting Information Available

Euler–Euler closure models; assessment of the multiphase operator splitting (MOS) in chemical and mass transfer regime; OCM microkinetic mechanism; mesh convergence analysis for the lab-scale reactor configuration; mesh convergence analysis for the industrial scale reactor configuration; sensitivity analysis of the specific surface area of the catalyst; numerical issues related to the linearization of the chemistry source term (PDF)

## Dedication

We dedicate this article to Enrico Tronconi in gratitude for his scientific mentorship at the occasion of his 65th birthday.
