# Supplementary material for: Coupling Euler–Euler and Microkinetic Modeling for the Simulation of Fluidized Bed Reactors: an Application to the Oxidative Coupling of Methane
Source: Ind Eng Chem Res. 2021 Feb 12;60(18):6687–97. doi: 10.1021/acs.iecr.0c05845 (PMC8154421; doi:10.1021/acs.iecr.0c05845)
Supplement: Supplementary file 1 — ie0c05845_si_001.pdf [file ie0c05845_si_001.pdf]

Supporting Information for

Coupling Euler-Euler and microkinetic modeling for the simulation of fluidized bed reactors: an application to the Oxidative Coupling of Methane

Daniele Micale[1], Riccardo Uglietti[1],
Mauro Bracconi[1] and Matteo Maestri[1]*

[1] Laboratory of Catalysis and Catalytic Processes, Dipartimento di Energia, Politecnico di Milano, via La Masa 34, 20156 Milano, Italy.

*to whom correspondence should be addressed:
matteo.maestri@polimi.it (M. Maestri)

Table of Contents

1 Euler-Euler Closure Models S2

2 Assessment of the Multiphase Operator Splitting (MOS) in Chemical and Mass Transfer Regime S3

3 OCM Microkinetic Mechanism S5

4 Mesh Convergency Analysis for the Lab-Scale Reactor Configuration S7

5 Mesh Convergency Analysis for the Industrial Scale Reactor Configuration S8

6 Sensitivity Analysis of the Specific Surface Area of the Catalyst S10

7 Numerical Issues related to the Linearization of the Chemistry Source Term S12

1 Euler-Euler Closure Models

Table S1– Closure models adopted in this work to describe the gas-solid drag force, the heat and mass transfer coefficients, the convective and diffusive fluxes and, the KTGF properties in the multiscale Euler-Euler framework

Quantity	Closure Model	Ref.
Gas-particle drag force	$\boldsymbol{F}_{gs} = \beta(\boldsymbol{U}_s - \boldsymbol{U}_g)$ $\beta = \begin{cases} 150\frac{\varepsilon_s\mu_g}{(1-\varepsilon_s)d_p^2} + \frac{7}{4}\frac{\varepsilon_s\rho_g\lvert\boldsymbol{U}_g - \boldsymbol{U}_s\rvert}{d_p} & \varepsilon_s > 0.2 \\ \frac{3}{4}C_D\frac{\varepsilon_s\rho_g\lvert\boldsymbol{U}_g - \boldsymbol{U}_s\rvert}{d_p}(1-\varepsilon_s)^{-1.65} & \varepsilon_s < 0.2 \end{cases}$ $C_D = \begin{cases} \frac{24}{\varepsilon_g Re_p}\left[1 + 0.15(\varepsilon_g Re_p)^{0.687}\right] & Re_p < 1000 \\ 0.44 & Re_p > 1000 \end{cases}$	Gidaspow[1]
Heat transfer coefficient, h	$h = \frac{Nu\lambda_g}{d_p}$ $Nu = (7 - 10\varepsilon_g + 5\varepsilon_g^2)\left(1 + 0.7Re_p^{0.2}Pr^{\frac{1}{3}}\right) + (1.33 - 2.4\varepsilon_g + 1.3\varepsilon_g^2)Re_p^{0.7}Pr^{\frac{1}{3}}$	Gunn[2]
Mass transfer coefficient, $K_{c,i}$	$K_{c,i} = \frac{Sh_i D_i}{d_p}$ $Sh_i = (7 - 10\varepsilon_g + 5\varepsilon_g^2)\left(1 + 0.7Re_p^{0.2}Sc_i^{\frac{1}{3}}\right) + (1.33 - 2.4\varepsilon_g + 1.3\varepsilon_g^2)Re_p^{0.7}Sc_i^{\frac{1}{3}}$	Gunn[2]
Conductive flux, $\boldsymbol{q}_{cond}$	$\boldsymbol{q}_{cond} = \lambda_g\nabla T_g$	Fourier[3]
Diffusive flux, $\boldsymbol{J}_i$	$\boldsymbol{J}_i = -\rho_g D_i\nabla x_{i,g}$	Fick[3]
Solid shear viscosity, μ_s	$\mu_s = \frac{4}{5}\varepsilon_s^2\rho_s d_p g_0(1+e)\sqrt{\frac{\theta}{\pi}} + \frac{2\frac{5\sqrt{\pi}}{96}\rho_s d_p\sqrt{\theta}}{g_0(1+e)}\left[1 + \frac{4}{5}g_0\varepsilon_s(1+e)\right]^2$	Gidaspow[1]
Solid bulk viscosity, λ_s	$\lambda_s = \frac{4}{3}\varepsilon_s^2\rho_s d_p g_0(1+e)\sqrt{\frac{\theta}{\pi}}$	Lun et al.[4]
Radial solid distribution, g_0	$g_0 = \left[1 - \left(\frac{\varepsilon_s}{\varepsilon_{s,max}}\right)^{\frac{1}{3}}\right]^{-1}$	Siclair & Jackson[5]
Solid conductivity, k_s	$k_s = \frac{2}{(1+e)g_0}\left[1 + \frac{6}{5}(1+e)g_0\varepsilon_s\right]^2\left(\frac{75}{384}\rho_s d_p\sqrt{\theta\pi}\right) + 2\varepsilon_s\rho_s d_p g_0(1+e)\sqrt{\frac{\theta}{\pi}}$	Gidaspow[1]
Dissipation of granular energy related to the inelastic particle-particle collisions, γ_s	$\gamma_s = 12(1-e^2)\frac{\varepsilon_s^2\rho_s g_0}{d_p\sqrt{\pi}}\theta^{\frac{3}{2}}$	Lun et al.[4]
Dissipation of granular energy related to the momentum exchange between the two phases, J_s	$J_s = \beta\left[3\theta - \frac{\beta d_p(\boldsymbol{U}_g - \boldsymbol{U}_s)^2}{4\varepsilon_s\rho_s\sqrt{\theta\pi}}\right]$	Louge at al.[6]

2 Assessment of the Multiphase Operator Splitting (MOS) in Chemical and Mass Transfer Regime

The Multiphase Operator Splitting approach has been tested in the chemical and mass transport regime in order to assess the validity of the method with different regimes. This assessment has been performed by considering a fixed bed reactor filled with particles having a diameter of 500 μm. A simple kinetic mechanism composed of a heterogeneous first-order irreversible reaction (Eq. (S1)-(S2)) has been employed in the analysis:

$$2A + B \rightarrow 2C \tag{S1}$$

$$r = k * C_A \tag{S2}$$

The operating conditions adopted for the numerical tests are a reactor temperature of 623.15 K, atmospheric pressure and a feed composition consisting of 0.015 v/v of A, 0.207 v/v of B and 0.778 v/v of inert. The computational domain is a cylinder (diameter = 0.01 m; height = 0.1 m) composed by 108,000 cells and the simulation has been performed by adopting a CFL equal to 0.2. The two regimes are obtained by changing the gas-to-solid transport coefficient to work at different Damkohler numbers (Da). In particular, a Da equal to 10^3 has been adopted to simulate the system operated in external mass transfer limited regime, while a Da equal to 10^{-2} has been selected as representative of the chemical regime.

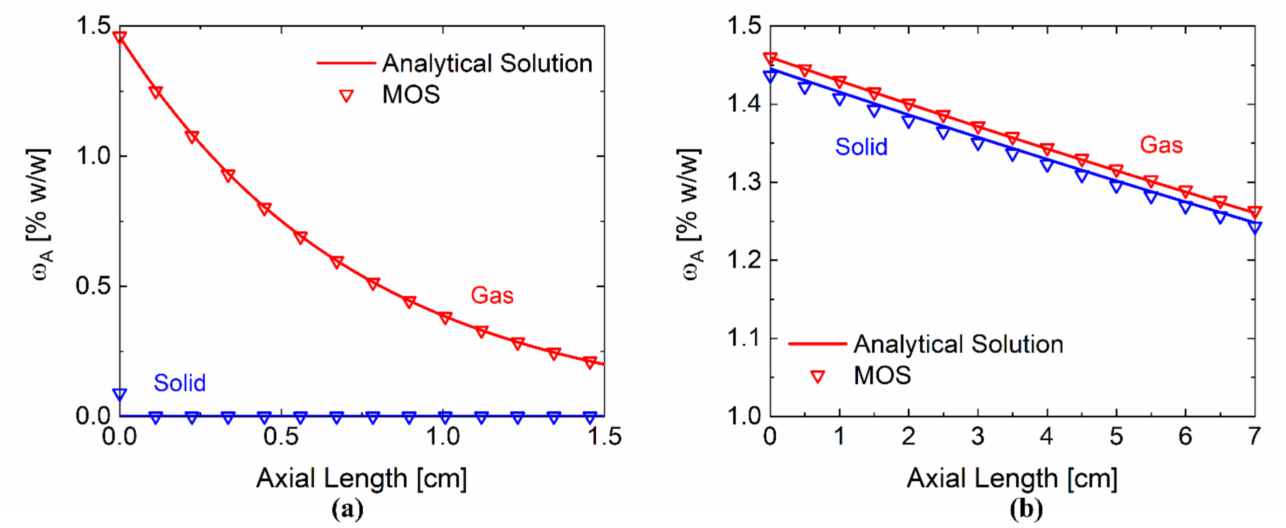

Figure S1 – Reactant mass fraction in the gas phase (red) and in the solid phase (blue) along the reactor length computed by means of analytical solution (straight line) and the MOS approach (open triangles) in a heterogeneous reaction operated in mass transfer regime (a) and chemical regime (b).

Figure S1 shows the trends of the reactant computed by means of analytical solution (eqs (S3)-(S4) for the mass transfer limited regime and eqs (S5)-(S6) for the chemical regime) and the MOS approach.

$$U_g * \frac{d\omega_{A,g}}{dL} = -\left(1 - \varepsilon_g\right) * K_{c.A} * S_V * \omega_{A,g} \tag{S3}$$

$$\omega_{A,s} = 0 \tag{S4}$$

$$U_g * \frac{d\omega_{A,g}}{dL} = -2 * \left(1 - \varepsilon_g\right) * k * \omega_{A,s} \tag{S5}$$

$$\omega_{A,s} = \frac{1}{1 + Da}\omega_{A,g} \tag{S6}$$

An excellent agreement has been obtained assessing the capability of the MOS to accurately predict the evolution of the system in both the conditions. Hence, the hereby proposed splitting method is able to deal with different regimes.

3 OCM Microkinetic Mechanism

The elementary steps composing the gas phase and heterogenous microkinetic schemes are reported in Table S2 and Table S3, respectively. The reaction rate is calculated by means of the modified Arrhenius equation:

$$r_i = k_i T^{\beta_i} \exp\left(\frac{E_i}{RT}\right) \qquad (S7)$$

where k_i is the temperature-independent pre-exponential coefficient of the *i-th* reaction, T is the temperature, β_i is the temperature exponent of the *i-th* reaction, E_i is the activation energy of the *i-th* reaction and, R is the ideal gas constant.

Table S2 – Elementary steps adopted to describe the gas-phase reactivity of the oxidative coupling of methane and corresponding parameter values[7,8]*.*

#	Reactions	k_i [s $^{-1}$ or cm/mol/s or cm^6/mol^2/s]	β_i [-]	E_i [kJ/mol]
1	$CH_4+O_2{=>}CH_3+HO_2$	$0.983\cdot10^{+13}$	0	193.86
2	$CH_4+H{=>}CH_3+H_2$	$0.234\cdot10^{+15}$	0	51.17
3	$CH_4+O{=>}CH_3+OH$	$0.127\cdot10^{+16}$	0	33.83
4	$CH_4+OH{=>}CH_3+H_2O$	$0.743\cdot10^{+15}$	0	41.43
5	$CH_4+HO_2{=>}CH_3+H_2O_2$	$0.401\cdot10^{+14}$	0	99.61
6	$CH_3+O_2{=>}CH_3O+O$	$0.308\cdot10^{+15}$	0	141.0
7	$CH_3+O_2{=>}CH_2O+OH$	$0.459\cdot10^{+14}$	0	103.66
8	$CH_3+HO_2{=>}CH_3O+OH$	$0.885\cdot10^{+14}$	0	0
9	$CH_3+CH_3+M{=>}C_2H_6+M$	$0.650\cdot10^{+20}$	0	0
10	$CH_3O+M{=>}CH_2O+H+M$	$0.258\cdot10^{+21}$	0	115. 0
11	$CH_2O+OH{=>}CHO+H_2O$	$0.580\cdot10^{+15}$	0	5.0
12	$CH_2O+HO_2{=>}CHO+H_2O_2$	$0.417\cdot10^{+13}$	0	40.12
13	$CH_2O+CH_3{=>}CHO+CH_4$	$0.7000\cdot10^{+14}$	0	25.03
14	$CHO+M{=>}CO+H+M$	$0.280\cdot10^{+16}$	0	64.360
15	$CHO+O_2{=>}CO+HO_2$	$0.171\cdot10^{+12}$	0	0
16	$CO+HO_2{=>}CO_2+OH$	$0.308\cdot10^{+15}$	0	107.34
17	$C_2H_6+H{=>}C_2H_5+H_2$	$0.910\cdot10^{+15}$	0	51.7
18	$C_2H_6+OH{=>}C_2H_5+H_2O$	$0.545\cdot10^{+15}$	0	17.16
19	$C_2H_6+CH_3{=>}C_2H_5+CH_4$	$0.239\cdot10^{+14}$	0	64.73
20	$C_2H_5+HO_2{=>}CH_3+CH_2O+OH$	$0.948\cdot10^{+13}$	0	0
21	$C_2H_5+M{=>}C_2H_4+H+M$	$0.596\cdot10^{+20}$	0	167.66
22	$C_2H_5+O_2{=>}C_2H_4+HO_2$	$0.635\cdot10^{+13}$	0	53.2
23	$C_2H_4+O_2{=>}C_2H_3+HO_2$	$0.281\cdot10^{+13}$	0	144.55
24	$C_2H_4+H{=>}C_2H_3+H_2$	$0.150\cdot10^{+15}$	0	42.7
25	$C_2H_4+OH{=>}C_2H_3+H_2O$	$0.612\cdot10^{+14}$	0	24.7
26	$C_2H_4+CH_3{=>}C_2H_3+CH_4$	$0.199\cdot10^{+12}$	0	51.46
27	$C_2H_4+OH{=>}CH_3+CH_2O$	$0.272\cdot10^{+13}$	0	0
28	$C_2H_3+M{=>}C_2H_2+H+M$	$0.121\cdot10^{+22}$	0	176.44
29	$C_2H_3+O_2{=>}C_2H_2+HO_2$	$0.5\cdot10^{+13}$	0	0
30	$C_2H_3+O_2{=>}CH_2O+CHO$	$0.55\cdot10^{+13}$	0	0
31	$C_2H_5+CH_3{=>}C_3H_8$	$0.8\cdot10^{+13}$	0	0
32	$C_3H_8+H{=>}C_3H_7+H_2$	$0.9\cdot10^{+15}$	0	32.0
33	$C_2H_4+CH_3{=>}C_3H_7$	$0.3\cdot10^{+12}$	0	29.0

34	C_3H_7=>C_3H_6+H	$0.15\cdot10^{+16}$	0	156.0
35	O_2+H=>OH+O	$0.22\cdot10^{+15}$	0	70.3
36	O_2+H+M=>HO_2+M	$0.139\cdot10^{+18}$	0	0
37	HO_2+HO_2=>O_2+OH+OH	$0.2\cdot10^{+13}$		0
38	H_2O_2+M=>OH+OH+M	$0.127\cdot10^{+18}$	0	199.36
39	C_2H_6=>C_2H_5+H	$0.4\cdot10^{+17}$	0	378.51
40	CH_3+OH=>CH_2+H_2O	$0.155\cdot10^{+7}$	2.1	10.0
41	CH_3+H=>CH_2+H_2	$0.182\cdot10^{+15}$	0	63.0
42	CH_2+O=>CH+OH	$0.912\cdot10^{+14}$	0	0
43	CH_2+OH=>CH+H_2O	$0.269\cdot10^{+12}$	0.7	108.0
44	CH_2+H=>CH+H_2	$0.400\cdot10^{+14}$	0	0
45	CH_2+C_2H_6=>CH_3+C_2H_5	$0.650\cdot10^{+13}$	0	33.1
46	CH+O=>CO+H	$0.400\cdot10^{+14}$	0	0
47	CH+O_2=>CHO+O	$0.100\cdot10^{+14}$	0	0
48	C_2H_2+O=>CH_2+CO	$0.410\cdot10^{+9}$	1.50	7.1

Table S3 – Elementary steps adopted to describe the heterogenous reactivity of the oxidative coupling of methane and corresponding parameter values[9].

#	Reactions	k_i [s^{-1} or cm/mol/s]	β_i [-]	E_i [kJ/mol]
1	O_2 + * => O_2*	$1.8\cdot10^{+7}$	0	0
2	O_2* => O_2 + *	$2.3\cdot10^{+13}$	0	163.176
3	O_2* + *=> O* + O*	$2.6\cdot10^{+23}$	0	104.6
4	O* + O* => O_2* +*	$2.6\cdot10^{+23}$	0	138.072
5	CH_4 + O* => CH_3 + OH*	$7.5\cdot10^{+8}$	0	36.98656
6	OH* + OH* => H_2O + O* + *	$2.6\cdot10^{+23}$	0	10.0416
7	CO_2 + * => CO_2*	$6.2\cdot10^{+8}$	0	0
8	CO_2* => CO_2 + *	$2.3\cdot10^{+13}$	0	190.70672
9	CO + O* => CO_2 + *	$4.7\cdot10^{+9}$	0	12.552
10	C_2H_4 + O* => C_2H_3 + OH*	$4.1\cdot10^{+8}$	0	24.6856
11	C_2H_5 + O* => C_2H_4 + OH*	$5.5\cdot10^{+7}$	0	0
12	C_3H_7 + O* => C_3H_6 + OH*	$6.1\cdot10^{+7}$	0	0
13	C_2H_6 + O* => C_2H_5 + OH*	$9.5\cdot10^{+9}$	0	41.84
14	CH_3 + O* => CH_2 + OH*	$1.9\cdot10^{+9}$	0	11.7152
15	CH_2 + O* => CH + OH*	$3.6\cdot10^{+11}$	0	49.7896
16	CH + O* => C + OH*	$8.9\cdot10^{+8}$	0	19.6648
17	C + O* => CO + *	$1.1\cdot10^{+11}$	0	0
18	CH_3 + O* => CH_3O*	$9.9\cdot10^{+8}$	0	0
19	CH_3O* + O* => CH_2O + OH* +*	$2.4\cdot10^{+23}$	0	0
20	CH_2O + O* => CHO + OH*	$3.4\cdot10^{+7}$	0	12.552
21	CHO + O* => CO + OH*	$6.9\cdot10^{+7}$	0	0

4 Mesh Convergence Analysis for the Lab-Scale Reactor Configuration

To show the effect of the mesh on the lab-scale configuration, a mesh convergency analysis has been performed. In particular, the analysis consists of simulating the lab-scale system by using three different grids characterized by a cell to particle ratio in the range 10-20. A ratio lower than 10 has not been simulated due to the not accurate KTGF predictions when a fine computational grid is adopted[1], while a ratio higher than 20 has not been tested due to the small number of cells in the cross section area that in our opinion is not suitable for accurate predictions. The size of the computational cells adopted to divide the lab-scale computational domain is smaller than the fluid dynamic structure present inside the units[10]. Therefore, the correlation proposed by Gidaspow[1] can be accurately used to describe the gas-solid interactions. The analysis has been carried out in terms of average methane and ethane content in the catalytic bed.

Figure S2 shows the temporal trend of the volume averaged mass fraction of methane and ethane in the catalytic bed (Eq (21) of the manuscript) obtained with the different computational grids.

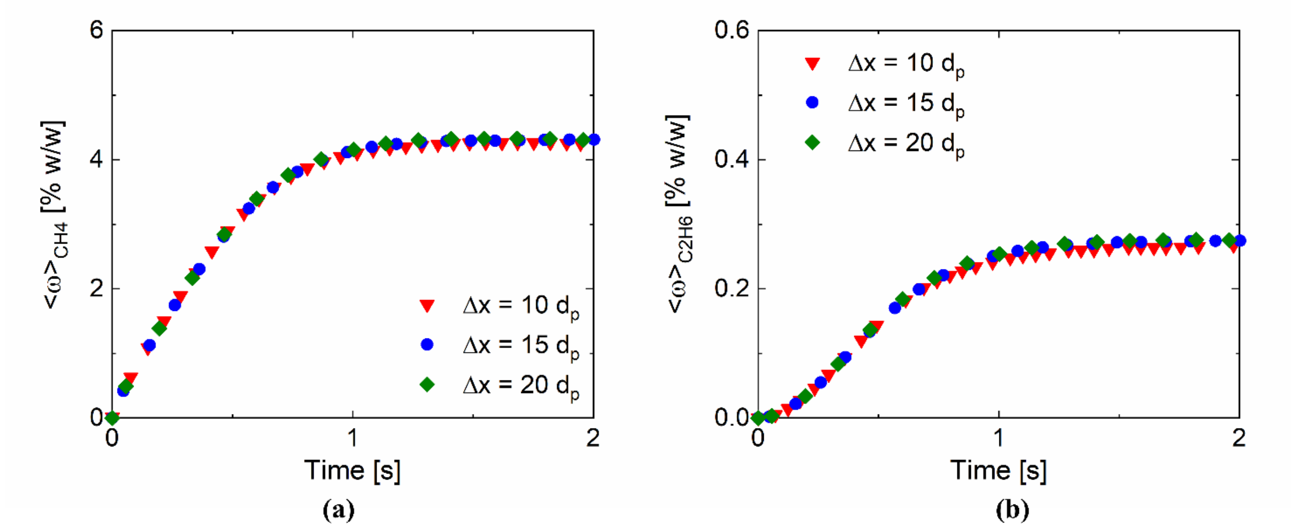

Figure S2 - Temporal trend of the volume average mass fraction of methane (a) and ethane (b) in the catalytic bed obtained with different computational domains in the lab-scale configuration.

The profiles of the two species show that the discretization has a negligible influence on the chemistry. In particular, a maximum deviation lower than 2% is obtained by using the three different computational grids both during the transient and at steady state. Consequently, the computational domain with a cell size of 10 dp can be properly adopted to validate the predictions of the MOS-based Euler-Euler framework against the experimental data.

5 Mesh Convergence Analysis for the Industrial Scale Reactor Configuration

To show the effect of the mesh on the industrial scale configuration, a grid convergency analysis has been performed. In particular, the computational domain has been divided with three different computational grids characterized by a maximum cell to particle ratio in the range 250-750. Figure S3 shows the maps of the steady-state void fraction obtained with the three different computational grids by considering the Gidaspow[1] model for the drag force.

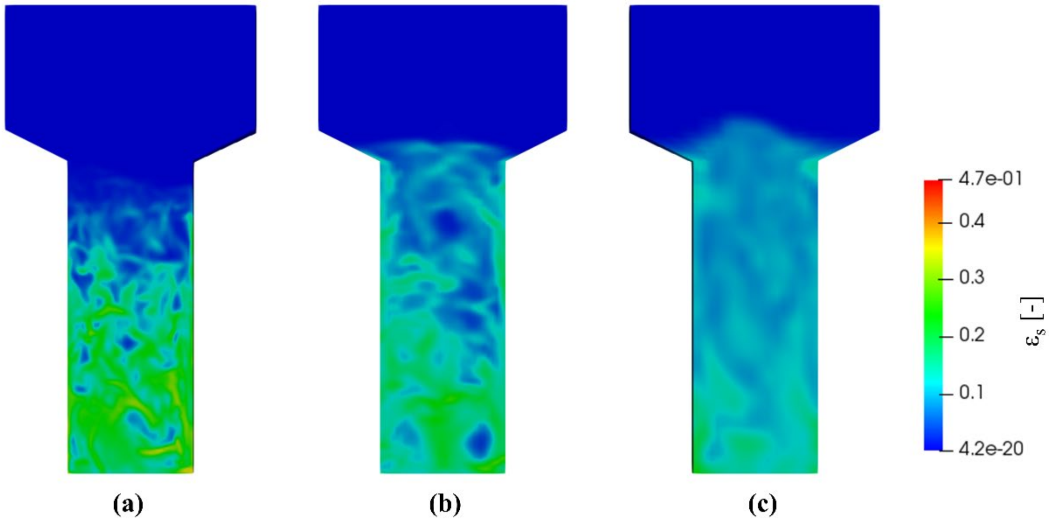

Figure S3 - 2D slices of the steady-state solid fraction obtained by adopting a computational grid having maximum cell size equal to 250 d_p (a), 500 d_p (b) and 750 d_p (c).

Figure S3 reveals that the fluid dynamic predictions of the Euler-Euler framework are strictly related to the computational grid adopted to divide the domain in these operating conditions, despite the same macroscopic regime (i.e. fast fluidization) obtained in the three simulations. This effect is related to the correlation adopted to describe the gas-particles interactions, which is not able to account for the effects of the cluster of particles[10] on the evaluation of the drag forces. This occurs when large cells (e.g. higher than 100 d_p) are considered. In fact, the loss of the discrete nature of the solid phases which characterizes the Euler-Euler model leads to the loss of the information related to the particles position and thus of the local flow structures (e.g. clusters of particles). This information is substituted by the average solid fraction content in the computational cell leading to an overestimation of the gas-particles interactions. Thus, taller fluidized beds are predicted by increasing the dimension of the computational cells. Different strategies have been proposed in literature to solve this problem. On the one hand, McKeen & Pugsley[11] artificially modified the drag forces by adopting an empirical corrective coefficient. On the other hand, the EMMS modeling approach[12] has been developed to

formulate an interaction model accounting for the flow structure loss. However, both the proposed approaches are not flexible, and they must be properly formulated to correctly represent the fluid dynamic of the investigated system.

Nevertheless, the chemical prediction obtained in the three computational domains are characterized by similar behavior of the systems (deviations lower than 2% in the pseudo steady-state), as shown in Figure S4, since the same fluidization regime is obtained in the three simulations. Consequently, since the aim of the showcase 2 is to show the capability of the MOS approach to deal with detailed chemistry and the long dynamic of industrial units, and this work is out from design purposes, we decide to adopt the computational grids having maximum cell size equal to 500 d_p. However, the MOS-based Euler-Euler framework can be employed with different correlations to describe the gas-particles interactions. Thus, the optimal correlation can be chosen to carry the validation or design of an industrial unit.

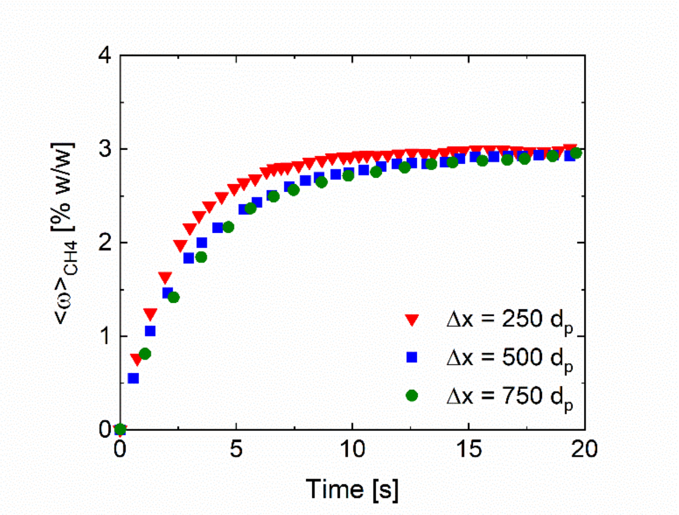

Figure S4 - Temporal trend of the volume average methane mass fraction in the catalytic bed obtained with different computational grids in the industrial scale configuration.

6 Sensitivity Analysis of the Specific Surface Area of the Catalyst

The sensitivity analysis of the specific surface area of the catalyst has been performed by increasing and decreasing the value adopted in the simulation of the manuscript of 5% and 10%. The lab-scale configuration has been selected to perform these simulations by setting a reactor temperature equal to 1023.15 K and a feed composition consisting of 0.1 v/v of methane, 0.04 v/v of oxygen and 0.86 v/v of nitrogen.

Figure S5 shows the temporal trend of the volume averaged methane mass fraction in the catalytic bed (eq (21) of the manuscript) obtained with the different specific surface area of the catalyst.

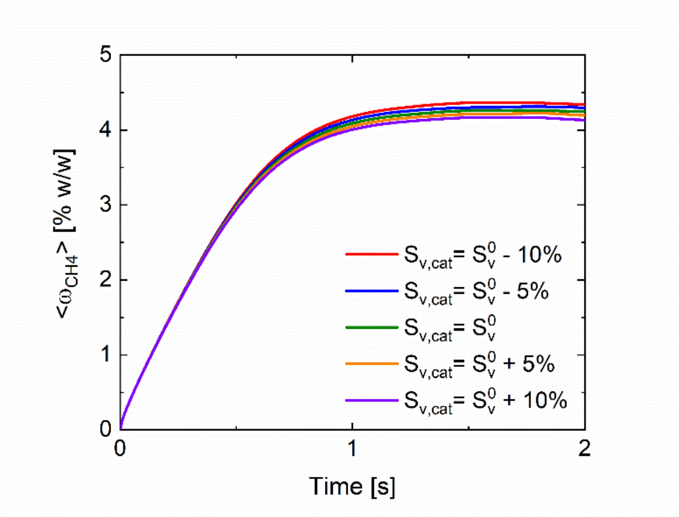

Figure S5 - Temporal trend of the volume average methane mass fraction in the catalytic bed obtained with different specific surface area of the catalyst $S_{v,cat}$ where S_v^0 =1.4487·10^{+5} m$^2_{cat}$/m$^3_{cat}$.

The decrement of the specific surface area of the catalyst leads to an increment of the volume average methane mass fraction due to the reduction of the catalyst activity. Coherently, the increment of the specific surface area of the catalyst leads to a decrement of the volume average methane mass fraction, as expected.

Table S4 – Maximum absolute deviation obtained by changing the specific surface area of the catalyst.

Variation	Maximum absolute deviation [%]
- 10%	2.45
- 5%	1.55
5%	1.24
10%	2.46

Table S4 reports the maximum absolute deviation with respect to the base case (i.e. S_v^0) for each change of the catalytic loading. As shown in the table, a maximum deviation lower than 2.5% has been obtained even by modifying the specific surface area of the catalyst by 10%. Therefore, the

overall effect of the catalytic loading is sub-linear and thus, the adopted value is robust and representative of the system.

7 Numerical Issues related to the Linearization of the Chemistry Source Term

The comparison between the MOS approach and the linearization of the chemistry source term performed in the lab-scale reactor revealed the presence of a delay in the reactor dynamics in the latter case. We also observed that the delay is a function of the time step and it is the result of numerical artifacts introduced by the linearization of the chemistry source terms. We ascribe the numerical delay to the implicit treatment of the linearized source term required to obtain a numerically stable reactive simulation. This approach employs a Taylor expansion as reported in eq (S8):

$$R_i(\omega_i(t_{n+1})) = R_i(\omega_i(t_n)) + \left.\frac{\partial R_i}{\partial \omega_i}\right|_{\omega_i(t_n)} \cdot (\omega_i(t_{n+1}) - \omega_i(t_n)) \qquad (S8)$$

where ω_i is the *i-th* species mass fraction, $R_i(\omega_i(t_n))$ is the *i-th* species production rate related to the *i-th* species mass fraction at the beginning of the time step and $R_i(\omega_i(t_{n+1}))$ is the *i-th* species production rate related to the *i-th* species mass fraction at the end of the time step. In doing so, the derivative term during a time step is considered constant and equal to the derivative term at the beginning of the time step when the maximum concentration of reactant is experienced. Since the derivative of the reaction rate with respect to the reactant is negative, the reaction rate experiences during a long time step an excessive decrement since the derivative is based on the maximum reactant condition. Consequently, a lower average reaction rate is experienced during the time step introducing the delay in the dynamics of the species. Coherently, the longer is the time step, the more relevant is the error and delay introduced since the less adequate is the linear reaction rate approximation.

References

(1) Gidaspow, D. *Multiphase Flow and Fluidization: Continuum and Kinetic Theory Descriptions*; Academic Press, 1994.

(2) Gunn, D. J. Transfer of Heat or Mass to Particles in Fixed and Fluidised Beds. *Int. J. Heat Mass Transf.* **1978**, *21* (4), 467–476.

(3) Bird, R. B.; Stewart, W. E.; Lightfoot, E. N. *Transport Phenomena*, 2nd ed.; John Wiley & Sons, I., Ed.; 2007.

(4) Lun, C. K.; Savage, S. B.; Jefferey, D. J.; Chepurniy, N. Kinetic Theories for Granular Flow: Inelastic Particles in Couette Flow and Slightly Inelastic Particles in a General Flowfield. *J. Fluid Mech.* **1984**, *140*, 223–256.

(5) Sinclair, J. L.; Jackson, R. Gas-particle Flow in a Vertical Pipe with Particle-particle Interactions. *AIChE J.* **1989**, *35* (9), 1473–1486.

(6) Louge, M. Y.; Mastorakos, E.; Jenkins, J. T. The Role of Particle Collisions in Pneumatic Transport. *J. Fluid Mech.* **2006**, *231*, 345–359.

(7) Sun, J.; Thybaut, J. W.; Marin, G. B. Microkinetics of Methane Oxidative Coupling. *Catal. Today* **2008**, *137* (1), 90–102.

(8) Zanthoff, H.; Baerns, M. Oxidative Coupling of Methane in the Gas Phase. Kinetic Simulation and Experimental Verification. *Ind. Eng. Chem. Res.* **1990**, *29* (1), 2–10.

(9) Simon, Y.; Baronnet, F.; Marquaire, P. M. Kinetic Modeling of the Oxidative Coupling of Methane. *Ind. Eng. Chem. Res.* **2007**, *46* (7), 1914–1922.

(10) Wang, W.; Lu, B.; Zhang, N.; Shi, Z.; Li, J. A Review of Multiscale CFD for Gas-Solid CFB Modeling. *Int. J. Multiph. Flow* **2010**, *36* (2), 109–118.

(11) McKeen, T.; Pugsley, T. Simulation and Experimental Validation of a Freely Bubbling Bed of FCC Catalyst. *Powder Technol.* **2003**, *129* (1–3), 139–152.

(12) Hansen, K. G.; Ibsen, C. H.; Solberg, T.; Hjertager, B. H. Eulerian/Eulerian CFD Simulation of a Cold Flowing FCC Riser. *Int. J. Chem. React. Eng.* **2003**, *1* (1). https://doi.org/10.2202/1542-6580.1032
